# Comparing constitutive and induced costs of symbiont-conferred resistance to parasitoids in aphids

**DOI:** 10.1002/ece3.491

**Published:** 2013-02-13

**Authors:** Christoph Vorburger, Pravin Ganesanandamoorthy, Marek Kwiatkowski

**Affiliations:** 1 Institute of Integrative Biology, ETH ZürichUniversitätstrasse 16, 8092, Zürich, Switzerland; 2 EAWAG, Swiss Federal Institute of Aquatic Science and TechnologyÜberlandstrasse 133, 8600, Dübendorf, Switzerland

**Keywords:** *Aphis fabae*, *Hamiltonella defensa*, *Lysiphlebus fabarum*, parasitoid, resistance, symbiosis

## Abstract

Host defenses against parasites do not come for free. The evolution of increased resistance can be constrained by constitutive costs associated with possessing defense mechanisms, and by induced costs of deploying them. These two types of costs are typically considered with respect to resistance as a genetically determined trait, but they may also apply to resistance provided by ‘helpers’ such as bacterial endosymbionts. We investigated the costs of symbiont-conferred resistance in the black bean aphid, *Aphis fabae* (Scopoli), which receives strong protection against the parasitoid *Lysiphlebus fabarum* from the defensive endosymbiont *Hamiltonella defensa*. Aphids infected with *H. defensa* were almost ten times more resistant to *L. fabarum* than genetically identical aphids without this symbiont, but in the absence of parasitoids, they had strongly reduced lifespans, resulting in lower lifetime reproduction. This is evidence for a substantial constitutive cost of harboring *H. defensa*. We did not observe any induced cost of symbiont-conferred resistance. On the contrary, symbiont-protected aphids that resisted a parasitoid attack enjoyed increased longevity and lifetime reproduction compared with unattacked controls, whereas unprotected aphids suffered a reduction of longevity and reproduction after resisting an attack. This surprising result suggests that by focusing exclusively on the protection, we might underestimate the selective advantage of infection with *H. defensa* in the presence of parasitoids.

## Introduction

The constant threat of infection by parasites and pathogens requires a significant investment in defense by most organisms. The rapidly expanding field of ecological immunology is concerned with understanding the ecology and evolution of immune defenses in a general life-history framework (Sheldon and Verhulst 1996). A central assumption of ecological immunology is that defenses against parasites are associated with costs. It is useful to distinguish two types of costs. The constitutive or standing costs are costs of having the ability to resist, that is, of possessing a particular type of immune defense. These costs are borne irrespective of whether an organism is parasitized or not, and can thus select against resistance in the absence of parasites (Schmid-Hempel 2003). The induced or actual costs are only incurred when a defense is indeed deployed upon contact with a parasite.

Both types of costs have been investigated in insects (Kraaijeveld et al. 2002). The existence of induced costs is generally undisputed (Schmid-Hempel 2005). They can be measured by the application of antigenic challenges that trigger an immune response, but do not lead to infection. Such manipulations have been shown to result in measurable reductions in insect longevity (Moret and Schmid-Hempel 2000; Armitage et al. 2003). Constitutive costs are typically investigated either by estimating genetic correlations between resistance to parasites and other components of fitness or by measuring correlated responses to selection for increased resistance. Such studies provided more ambiguous results (e.g. Gwynn et al. 2005; von Burg et al. 2008), although some clearly supported the existence of constitutive costs of resistance (Schmid-Hempel 2011; ch. 5.2.3). For example, lines of *Drosophila melanogaster* selected for increased resistance to the parasitoid *Asobara tabida* suffer from reduced competitive ability at the larval stage (Kraaijeveld and Godfray 1997).

The issue of defense costs also arises if the hosts' own defenses against parasites are supplemented by resistance-conferring microbial symbionts. The best known examples include *Spiroplasma* protecting *D. neotestacea* against parasitic nematodes (Jaenike et al. 2010), *Wolbachia* protecting *D. melanogaster* against RNA viruses (Hedges et al. 2008; Teixeira et al. 2008), or – the system of concern here – endosymbiotic bacteria protecting aphids against parasitoids (Oliver et al. 2003). Although natural populations of aphids do exhibit genetic variation for resistance to parasitoids (Henter and Via 1995; von Burg et al. 2008; Sandrock et al. 2010), even more variation is explained by the presence or absence of the maternally transmitted endosymbiotic bacterium *Hamiltonella defensa* (Oliver et al. 2005; Vorburger et al. 2009). This facultative endosymbiont occurs in multiple species of aphids and provides strong protection by expressing phage-encoded toxins that kill the egg or early larval stages of hymenopteran parasitoids (Oliver et al. 2009). Despite this obvious benefit, *H. defensa* is not fixed in aphid populations and typically occurs at intermediate frequencies (Simon et al. 2003; Oliver et al. 2006; Vorburger et al. 2009), suggesting that symbiont-conferred resistance comes at a constitutive cost associated with harboring *H. defensa*. This is supported by work on pea aphids, *Acyrthosiphon pisum*, showing that aphids infected with *H. defensa* are outcompeted by uninfected aphids in the absence of parasitoids (Oliver et al. 2008), and suffer reductions in several correlates of fitness (Simon et al. 2011). Work on black bean aphids, *Aphis fabae*, further showed that infection with *H. defensa* curtails aphid lifespan, resulting in a reduced lifetime reproduction when parasitoids are absent (Vorburger and Gouskov 2011). Computer simulations of a mathematical model showed that the right balance between benefits (i.e. protection against parasitoids) and constitutive costs of harboring defensive symbionts can indeed maintain coexistence between infected and uninfected aphids, yet the parameter space enabling coexistence was extremely narrow (Kwiatkowski and Vorburger 2012). The parameter space for coexistence expanded, however, when induced costs of symbiont-conferred protection were introduced in the model in addition to constitutive costs (Kwiatkowski and Vorburger 2012). Such induced costs could occur, for example, if the symbiont's means of protection (toxin production in the case of *H. defensa*) are upregulated upon contact with parasites and have negative effects on the host as well as the parasite.

The theoretical result mentioned above provided the motivation for the experiment reported here, in which we jointly estimated induced and constitutive costs of symbiont-conferred resistance in a well-studied aphid–parasitoid system. This experiment clearly retrieved the expected constitutive costs of symbiont-conferred resistance, but it did not provide any evidence for an induced cost. In fact, symbiont-protected aphids benefitted from resisting a parasitoid attack relative to symbiont-protected controls that were not attacked.

## Materials and methods

### Insects

**Figure 1:**
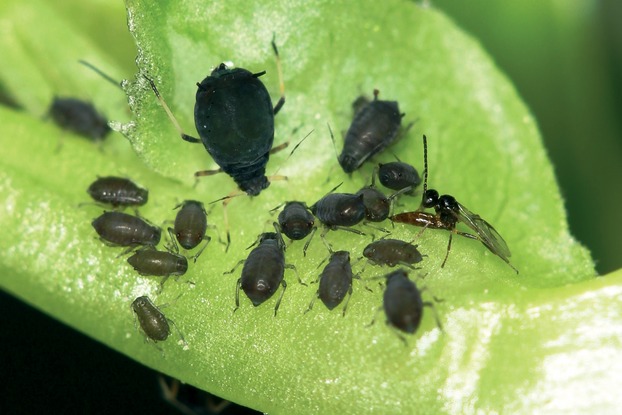
An adult female black bean aphid (*Aphis fabae*) and several of her clonal offspring under attack a by an ovipositing female of the aphid parasitoid *Lysiphlebus fabarum*. Photograph by Christoph Vorburger.

The black bean aphid, *Aphis fabae* (Fig. 1), is among the most abundant aphids in the temperate regions of the northern hemisphere and an important pest of broad bean (*Vicia faba*) and different beet crops (*Beta* sp.) (Blackman and Eastop 2000). As a cyclical parthenogen, it has many viviparous, asexual generations throughout the growth season, followed by one sexual generation in late autumn that produces frost-resistant, overwintering eggs. Based on a survey of Swiss and French populations, approx. 50% of *A. fabae* individuals harbor *H. defensa* (R. Rouchet & C. Vorburger, unpubl. data).

*Aphis fabae* is one of the main hosts of the aphid parasitoid *Lysiphlebus fabarum* (Hymenoptera: Braconidae: Aphidiinae; Fig. 1) (Starý 2006), which reproduces by thelytokous parthenogenesis in most populations (Belshaw et al. 1999; Starý 1999; Sandrock and Vorburger 2011). This enables the use of genetically homogeneous all-female lines of both antagonists in experiments. After oviposition of a single egg by the female parasitoid, the larva hatches and develops inside the still active aphid. After a period of growth, the larva kills the aphid, spins a cocoon inside the dead host's exoskeleton and pupates. This stage is called a ‘mummy’, from which the adult wasp emerges after metamorphosis.

For the experiment, we used a single clone of *A. fabae* (nr. 405), which was collected in July 2006 at St. Margrethen in north-eastern Switzerland. It was maintained since then on seedlings of broad bean (*V. faba*) at 18–20°C and a photoperiod of 16 h. Under these conditions, *A. fabae* reproduces by parthenogenesis exclusively. Based on a screening with general bacterial primers as well as symbiont-specific primers for the 16S rRNA gene, clone 405 is naturally uninfected with any known facultative endosymbionts of aphids (Vorburger et al. 2009). For an unprotected clone, it exhibits a relatively low susceptibility to *L. fabarum* (35% parasitism in a standard assay; Vorburger et al. 2009). The latter is important because for the present experiment, we required individuals that resisted parasitism by *L. fabarum* without protection by *H. defensa*. In March 2009, we generated a *H. defensa*-infected line of clone 405 by microinjection of hemolymph from an infected donor clone (Chen and Purcell 1997). The donor clone (nr. 76) was collected in May 2006 near La Grande Motte in southern France. The new line was labeled 405^H76^. It maintained a stable, heritable infection with *H. defensa* from clone 76, which we confirmed by diagnostic PCR using the primers published in Ferrari et al. (2012) immediately before the experiment described below. An earlier experiment has shown that this isolate of *H. defensa* provides a high level of protection against *L. fabarum* (Schmid et al. 2012).

As parasitoids, we used a parthenogenetic line of *L. fabarum* labeled 07-64, founded by a single female collected in September 2007 from a colony of *A. fabae* at Wildberg near Zürich, Switzerland. It was maintained in the laboratory on a *H. defensa*-free clone of *A. fabae* since its collection. Because they had been in culture for several years since their collection, the identity of the aphid and parasitoid lines used was verified by microsatellite genotyping 2 months before setting up the present experiment.

### Experimental design

The basic design of the experiment was to compare components of fitness between a control group that was not attacked by parasitoids and a treatment group that was attacked but resisted parasitism for both aphid sublines (405 and 405^H76^). The comparison between the two control groups was then used to assess constitutive costs of symbiont-conferred resistance. If in the absence of parasitoids, individuals possessing *H. defensa* are less fit than genetically identical individuals without *H. defensa*, we interpret this as evidence for a constitutive cost of possessing this defensive symbiont. The comparison between treatment groups is used to assess induced costs. A fitness loss after resisting the parasitoid is expected and may simply reflect the negative effects of the stab or the venom that female parasitoids inject with their egg (Beckage and Gelman 2004; Vorburger et al. 2008). In itself, this would not provide evidence for induced costs of resistance. But if this fitness loss is more severe in aphids with *H. defensa* than in those without, the difference can be interpreted as evidence for an induced deployment cost of symbiont-provided defense.

### Experimental Procedures

To avoid any confounding of symbiont-related differences between lines 405 and 405^H76^ by environmental maternal effects carried over from the stock culture, each line was split into 12 sublines reared on separate plants for one generation prior to the experiment. When the aphids of these sublines were adult, we transferred four females per subline to new plants and allowed them to reproduce for 24 h before discarding them. Two days later, when the offspring were 48- to 72-h old (mostly 2nd instar nymphs), they were exposed to parasitoids. For this, we clipped plant parts containing approx. 10–15 nymphs and placed them in small petri dishes (3.5 cm diameter). A single female of *L. fabarum* was then added to the dish and monitored continuously. When we observed a parasitoid attack (oviposition attempt consisting of curling the abdomen forward and stabbing the aphid), the attacked aphid was removed immediately, assigned to the treatment group and placed individually on a new seedling growing in a 0.07 l pot and covered with a cage. When approximately two-thirds of the aphids had been attacked, the remaining individuals were assigned to the control group and also caged individually on new plants. The advantage of this procedure was that the control aphids experienced exactly the same conditions as the treatment group. For example, they also perceived the presence of parasitoids, which is important because this may in itself trigger a life-history response. The disadvantage of this procedure was that the control aphids were not a truly random sample. They were those individuals that the parasitoids would have attacked last within the batches of 10–15 aphid nymphs we exposed to them. If the parasitoids showed some preference in the order they attacked their hosts, this could have introduced a bias. However, this problem should have been minor, because the host batches were very homogeneous (genetically identical individuals born on the same day), and because this potential bias would have applied to both aphid lines used.

The production of aphid nymphs and their exposures to parasitoids were temporally staggered over 2 days to obtain a sufficient number of stabbed individuals, but we took care to treat approximately equal numbers of individuals from both lines on each day. Overall, we obtained 110 aphids that suffered a single parasitoid attack (57 for line 405 and 53 for line 405^H76^) and 49 aphids that were not attacked (25 for line 405 and 24 for line 405^H76^). The individually caged aphids were placed on random positions in a large plastic tray on an illuminated shelf in a climatized room at 20°C. We checked the aphids once per day and recorded the time until adult ecdysis (development time) and adult mass, measured to the nearest microgram on a MX5 microbalance (Mettler Toledo GmbH, Greifensee, Switzerland) on the first day an individual was found adult. Nine days after exposure to parasitoids, all individuals from the treatment group that were parasitized successfully were clearly recognizable as mummies and discarded. None of the mummified aphids had produced any offspring before being killed by the parasitoid. We then transferred all survivors to new plants and counted their offspring on the old plants. This procedure was repeated every 5 days thereafter until the aphids' death. We recorded the time until death (checked daily) as well as the total number of offspring the aphids produced (lifetime reproduction). Over the course of the many transfers during the experiment, seven individuals were accidentally killed or lost and had to be omitted from the analyses of some or all traits (depending on when they were lost).

### Statistical analyses

We analyzed the survival data with a Cox proportional hazards regression in R 2.14.1 (R Development Core Team 2011), testing for the effects of aphid line, treatment, and their interaction. Development time, adult mass, and lifetime reproduction were analyzed with linear models in SPSS 19 (IBM Corp. NY, USA), testing for the same effects. The 2 days on which exposures to parasitoids took place were initially treated as experimental blocks in the analyses, but because this effect was far from significant in all analyses, we pooled the block variance into the residual.

## Results

**Figure 2:**
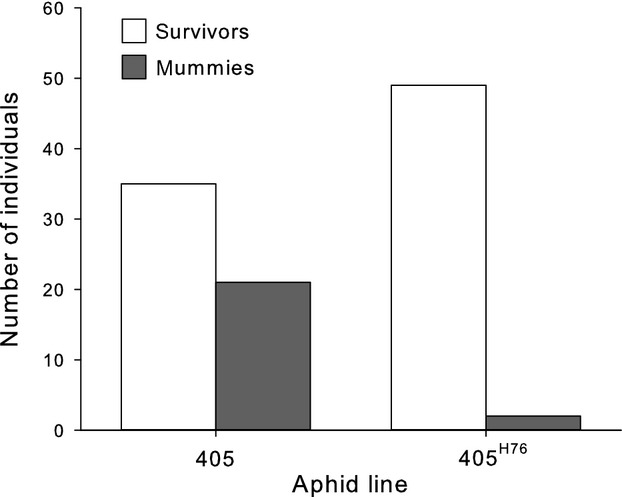
Increased aphid resistance to parasitoids provided by the defensive endosymbiont *Hamiltonella defensa*. Bars depict the numbers of black bean aphids (*Aphis fabae*) that were successfully parasitized and killed by the parasitoid *Lysiphlebus fabarum* (mummies) and the numbers of individuals that resisted the parasitoid (survivors) for two genetically identical lines that did (405^H76^) or did not (405) harbor *H. defensa*.

In the treatment group of aphid line 405, a total of 21 individuals (38%) were mummified and thus successfully parasitized by *L. fabarum*. Only two of the attacked individuals were mummified in the treatment group of line 405^H76^ (4%). This difference is significant (χ^2^ = 17.83, df = 1, *P* < 0.001) and illustrates the strong protection against parasitoids provided by *H. defensa* (Fig. 2).

**Figure 3:**
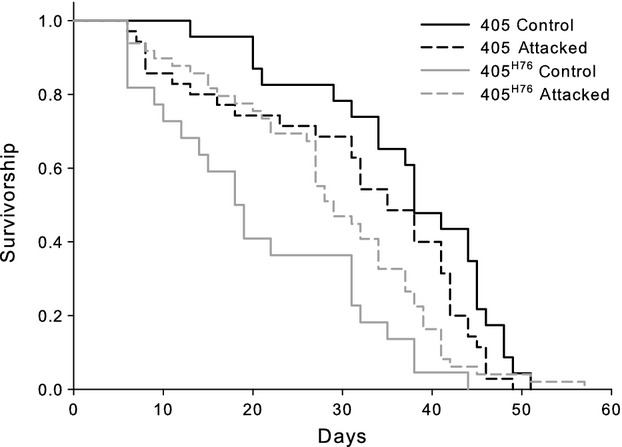
Survivorship curves of the control and treatment groups from aphid lines 405 (uninfected with *Hamiltonella defensa*) and 405^H76^ (harboring *H. defensa*). The treatment groups resisted a single attack by the parasitoid *Lysiphlebus fabarum*, the control groups were not attacked.

The survivorship curves of both aphid lines' control groups and the survivors from the treatment groups (individuals that were attacked but not mummified) are illustrated in Figure 3. Comparing the two control groups reveals the expected longevity cost of harboring *H. defensa*. When not attacked by parasitoids, uninfected aphids lived longer on average than genetically identical aphids harboring *H. defensa* (Fig. 3). This difference is mainly responsible for the significant line effect in the Cox regression (LR χ^2^ = 11.78, df = 1, *P* < 0.001). Unexpectedly, resisting a parasitoid attack had opposite effects on longevity in the two lines. Aphids without *H. defensa* died at a younger age relative to the control group after surviving a parasitoid attack, whereas aphids with *H. defensa* lived longer on average after resisting an attack (Fig. 3). This is reflected in a non-significant treatment effect (χ^2^ = 0.38, df = 1, *P* = 0.536), but a significant aphid line × treatment interaction (χ^2^ = 7.40, df = 1, *P* = 0.005).

**Figure 4:**
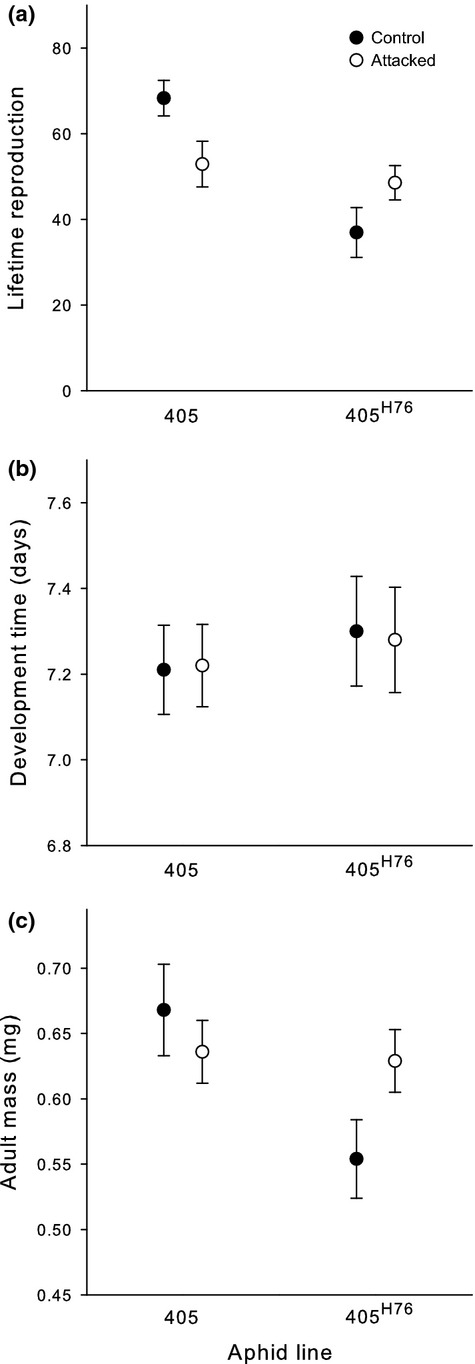
Life-history traits of the control and treatment groups from aphid lines 405 (uninfected with *Hamiltonella defensa*) and 405^H76^ (harboring *H. defensa*): (a) lifetime number of offspring, (b) development time from birth to adult ecdysis, and (c) adult mass on the day of adult ecdysis. The attacked groups resisted a single attack by the parasitoid *Lysiphlebus fabarum*, the control groups were not attacked. Error bars depict ±1 SE.

The longevity differences between groups translated into a similar pattern for lifetime reproduction (Fig. 4a). Overall, *H. defensa*-infected aphids produced fewer offspring over their lifetime and we also observed a significant aphid line × treatment interaction (Table 1, Fig. 4a). The uninfected aphids incurred a reduction in their lifetime reproduction when they resisted a parasitoid attack, whereas the attacked *H. defensa*-infected aphids exhibited an increased lifetime reproduction relative to controls.

**Table 1:** General linear model results for the life-history traits measured

Source of variation	df	MS	*F*	*P-*value
Development time				
Aphid line	1	0.178	0.392	0.532
Treatment	1	0.001	0.001	0.970
Aphid line × treatment	1	0.005	0.010	0.920
Residual	123	0.453		
Adult mass				
Aphid line	1	0.104	4.311	0.040
Treatment	1	0.014	0.573	0.451
Aphid line × treatment	1	0.081	3.351	0.070
Residual	123	0.024		
Lifetime reproduction				
Aphid line	1	9248.068	12.043	0.001
Treatment	1	104.351	0.136	0.713
Aphid line × treatment	1	5280.694	6.877	0.010
Residual	125	767.926		

We also compared development time (age at adult ecdysis) and adult mass among the four groups (Table 1, Fig. 4b and c). The two aphid lines did not differ significantly in their development time, nor did the treatment have a significant effect, but note that development time was measured with very coarse resolution (1-day intervals), such that small differences would have remained undetected. For adult mass, on the other hand, we detected a marginally significant effect of aphid line and a marginally non-significant line × treatment interaction (Table 1). The uninfected aphids were heavier on average with little difference between the treatment and control groups, whereas the *H. defensa*-infected aphids were lighter overall, but exhibited a marked difference between treatment groups (Fig. 4c). Also for this trait, surviving a parasitoid attack tended to have a positive rather than a negative effect on aphids harboring *H. defensa*.

## Discussion

The goal of this study was to estimate and compare constitutive as well as induced costs of symbiont-conferred resistance to parasitoids in a clone of the black bean aphid. A constitutive cost was clearly evident. In the absence of parasitoids, aphids harboring the protective symbiont *H. defensa* suffered from a reduced longevity and a lower lifetime reproduction compared with uninfected aphids of the same clone. This effect has been demonstrated previously for several isolates of *H. defensa* in two genetic backgrounds (Vorburger and Gouskov 2011) and was therefore expected. Quite unexpected was the result regarding potential induced costs of symbiont-conferred resistance. In aphid line 405, we found that even if an individual is able to prevent parasitoid development, the attack alone has negative effects in terms of reduced longevity and reproduction. This may be caused by the attack itself (lesion and venom injection), by the mounting of innate defenses, or by a combination of these effects. But in aphid line 405^H76^, the opposite was true. Not only did symbiont-protected aphids show near-complete resistance to *L. fabarum*, they also benefitted from increased longevity and lifetime reproduction following a parasitoid attack. Thus, we observed an induced benefit rather than an induced cost of symbiont-conferred resistance to parasitoids.

Some caveats are in order here. First, by isolating aphids immediately after being stabbed by the parasitoid, we constrained the number of attacks strictly to one. Aphid parasitoids tend to avoid superparasitism because only a single wasp can develop per aphid (e.g. Outreman et al. 2001), but multiple attacks may occur nevertheless in a natural situation. We cannot exclude the possibility that aphids protected by *H. defensa* would also start suffering from negative effects if forced to resist multiple attacks. Second, some of the parasitoid attacks we observed may not have resulted in oviposition. In an earlier study, we estimated that a minimum of 70% of single attacks observed by eye result in the injection of at least one egg (rarely two) (Vorburger et al. 2010). Some proportion of the survivors from the treatment groups may thus not have had to resist and suppress parasitoid development. However, this applies to both aphid lines and should not have biased our results, as there is no indication that aphid parasitoids are less likely to oviposit in resistant, symbiont-protected aphids (Henter and Via 1995; Oliver et al. 2003; Bensadia et al. 2006). A recent study on the parasitoid *Aphidius ervi* attacking pea aphids also found that parasitoids do not avoid ovipositing in aphids harboring *H. defensa* (Oliver et al. 2012). In fact, this study even suggested that parasitoids inject more eggs in symbiont-protected aphids to increase the probability of successful parasitism (Oliver et al. 2012). We do not know if this applies also to *L. fabarum* and we restricted the potential for superparasitism by allowing for single attacks only, but if anything, such a behavior would have biased the results against finding a benefit as we did here. A third caveat is related to the way we assigned individuals to control groups. If parasitoids were somehow able to assess the potential fitness of their hosts and attacked fitter individuals first, our control groups would have been biased toward less fit individuals (but note that batches of hosts were genetically identical and very similar in age). This would not change the fact that there were highly significant aphid line × treatment interactions, that is, opposite effects of parasitoid attack on longevity and reproduction in protected and unprotected aphids, respectively, but it could change their interpretation. One would then have to postulate that surviving a parasitoid attack has a strong negative effect on the fitness of uninfected aphids, but no negative effect on aphids harboring *H. defensa*. A final caveat is that only a single isolate of *H. defensa* in one genetic background was used in the present experiment. We know that the constitutive longevity cost of harboring *H. defensa* can be generalized as it was observed for multiple isolates (Vorburger and Gouskov 2011), but we cannot be sure that the induced effect observed here is a general phenomenon as well. This will require additional experiments with multiple genotypes of hosts, symbionts, and parasitoids.

Nevertheless, observing increased longevity and reproduction in symbiont-protected aphids that resisted a parasitoid attack is interesting and raises the question of the underlying mechanism. At present, we can only offer a speculative but testable hypothesis: attacks by parasitoids may reduce the symbiont's population size in the affected aphids. Parasitoids are under selection to evolve counteradaptations to host defenses and there is genetic variation in natural populations of parasitoids for the ability to overcome symbiont-conferred resistance (Rouchet and Vorburger 2012; Schmid et al. 2012). Indeed, an experimental evolution experiment using *A. ervi* and pea aphids has shown that parasitoids can evolve increased infectivity on *H. defensa*-protected aphids very rapidly (Dion et al. 2011b). How parasitoids adapt is currently unknown, but one possible mechanism is the injection of antimicrobial compounds at oviposition that suppress the host's population of defensive symbionts. Attacked aphids might thus harbor fewer defensive symbionts than aphids that were not attacked, at least temporarily. If the parasitoid larva fails to develop nevertheless, the host might actually benefit from the suppression of symbionts, because harboring *H. defensa* is associated with fitness costs resulting from reduced longevity (Vorburger and Gouskov 2011). Although entirely speculative at the moment, this hypothesis warrants further investigation. An alternative explanation could be that the harboring of protective symbionts entails a reduction in innate immune activity in aphids, such that they show a reduced response in case of parasitism. If innate resistance has induced costs, symbiont-protected aphids would save these costs, although it is difficult to see how this could translate into a benefit as observed here.

Independent of its mechanistic basis, the lack of an induced cost of defense conferred by symbionts or even a fitness increase upon resisting a parasitoid attack has consequences for understanding the prevalence of infection with such symbionts. Our model had suggested that induced costs of symbiont-conferred resistance in addition to constitutive costs could facilitate the coexistence of infected and uninfected hosts (Kwiatkowski and Vorburger 2012). Now it appears that at least for the *A. fabae*/*H. defensa* system, only the constitutive cost can be called upon, and that by focusing exclusively on the protection, we might even underestimate the selective advantage of infection with *H. defensa* when parasitoids are abundant.

Our result is also interesting in the light of a recent study by Dion et al. (2011a), showing that pea aphids reduce their behavioral defenses against parasitoids when infected with *H. defensa*. Because they enjoy strong physiological resistance conferred by the endosymbiont, they may economize on risky behavioral defenses such as dropping off the host plant (Dion et al. 2011a). However, this is only adaptive if a resistant aphid's fitness is not curtailed by strong induced costs of the protection by symbionts. Here, we found that this condition is fulfilled for a clone of the black bean aphid. Symbiont-protected aphids may indeed have little reason to run from parasitoids.
